# Role of a dentist in discrimination of abuse from accident

**DOI:** 10.4103/0974-2948.71049

**Published:** 2010

**Authors:** G Sujatha, G Sivakumar, T R Saraswathi

**Affiliations:** *Department of Oral Pathology, Sri Venkateswara Dental College and Hospital, Thalambur, Chennai, India*; 1 *Vishnu Dental College, Bhimavaram, Andhra Pradesh, India*

**Keywords:** Abuse, dentist, identification

## Abstract

Most physical injuries resulting from abuse or violence are found on the head and neck areas that are clearly visible to the dental team during examination. Given that dental professionals routinely assess the head, face, and neck of patients, they are in a unique position to identify the signs of abuse and neglect. Involvement of a dentist in the protection team may be beneficial and can lead to early intervention. This article brings to light the importance of questioning and physical examination and the role of a dentist in identifying abuse, assault, and accident.

## Introduction

Abuse and violence cross geographic and cultural boundaries and social and economic strata. It is common among the rich and the poor, the well-educated and the less so, the young and the middle-aged, city dwellers and the rural folk. It is a universal phenomenon.[1]

We dentists assess the head and neck routinely and have a great chance in identifying an abuse. Child abuse at Children’s Hospital Medical Center in Boston found that more than 65% of all the cases of physical abuse involved injuries to the head, neck, or mouth.[2]

### Why lack of recognition and intervention?

Although we have a chance of coming across a victim, usually it goes unnoticed due to the following reasons.

- Lack of training and experience in identifying
- Limited knowledge on how to intervene effectively
- Fear of litigation
- Spousal abuse rarely causes us to intervene
- The presence of a partner or children
- Concern about offending patients
- Embarrassment about bringing up the topic

It is important for every one of us to know that an abuse can happen anywhere and there is no social barrier. Most often the abuser is a well-known person to the victim and it is always wrong to stereotype the typical abuser. Abuse can be premeditated or deliberate, it can happen anywhere to anyone.

## Indicators of Abuse

Indicators are signs or clues that abuse has occurred.[3] These indicators can be physical, behavioral, and psychologic.

### Physical Indicators

Physical indicators are the most visible forms of abuse. They can be observed as any of the following.

- Sprains, dislocations, fractures, or broken bones
- Burns from cigarettes, appliances, or hot water
- Abrasions on arms, legs, or torso that resemble rope or strap marks
- Internal injuries evidenced by pain, difficulty with normal functioning of organs, and bleeding from body orifices
- Bruises

The following types of bruises are rarely accidental:

- Bilateral bruising to the arms (may indicate that the person has been shaken, grabbed, or restrained)
- Bilateral bruising of the inner thighs (may indicate sexual abuse)
- “Wrap around” bruises that encircle an older person’s arms, legs, or torso (may indicate that the person has been physically restrained)
- Multicolored bruises (indicating that they were sustained over time)
- Injuries healing through “secondary intention” (indicating that they did not receive appropriate care)
- Signs of traumatic hair and tooth loss.[3]
- Craniofacial, head, face, and neck injuries occur in more than half of the cases of child abuse.[4] Bruises on the cheek, back, chest, and upper arm are much less commonly observed but may be seen in infants as they begin to walk independently. Bruises on the abdomen, buttocks, hands, and feet are extremely rare in both infants and toddlers. Bruises that are not over bony prominences are uncommon.[5]

### Behavioral indicators

Behavioral indicators need some amount of attention and in this the injuries are usually unexplained or explanations are not convincing. The accompanying person provides different explanations about how the injury has occurred and there would be a history or evidence of similar injuries. There would be a delay between the time of injury and the time at which the person is brought in for care.

### Psychologic indicators

The psychologic indicators are avoidance, fear, anxiety, low self-esteem, and depression. The psychologic indicators are usually clearly visible and the victim is highly withdrawn.

## Points to Diagnose

Keeping these indicators in mind, the important points that would be helpful in diagnosis are

- Is the injury consistent with the given history or explanation of the cause.
- Is the explanation of the injury consistent with the physical findings?
- Is there evidence of previous or repeated trauma?
- Are there multiple skin lesions or bruises that are strongly suggestive of abuse.
- Does the parent or child show inappropriate behavior?
- Is there evidence of neglect or poor supervision?
- Is there hesitation in history taking as if wanting to say more?

## Discrimination of Abuse and Accident

Diagnosing suspected abuse or neglect is only the first step and dentists must be prepared to take an immediate remedial action on behalf of the victim.[6]

We dentists can identify abuse from clinical history and physical injuries

### Clinical history

When questioned about the nature and time of injury in case of abuse they are usually not very clear but in case of an accident it is very precise. The role of the accompanying person is very supportive and caring in case of an accident, whereas in case of abuse they are usually very interfering.

### Physical injuries

Physical injuries are the most visible form of abuse and may be defined as any act that results in a nonaccidental trauma. They are usually seen as any one or a combination of the following

- Bruises
- Burns
- Lacerations and abrasions
- Skeletal injuries
- Head Injuries
- Bite marks

Many of the indicators listed below can be explained by other causes and no single indicator can be taken as a conclusive proof. It is better to look for patterns or clusters of indicators that suggest a problem.

**Table:** 

Anatomical region	Observations[7]
Buttocks and lower back	Burns—cigarette, match tip/incense, ring shaped (stove),branding burns (heated metal)Slap marks Sexual abuse (anus)
Genitals and inner thighs	Bruising (possible sexual abuse)
Spine	Bruising on bony prominences, tenderness
Upper and lower limbs	Bowing, angulation near the joints, bruising on bony prominences
Pelvis	Bruising on bony prominences
Abdomen	Bruising, distention
Chest	Depressed sternum, flail chest, asymmetrical shape, flattened, bruising
Head	Swelling, prominent anterior fontanel, patchy hair loss
Forehead	Bruising
Eyes	Periocular bruising, intraocular hemorrhage
Cheeks	Slap marks, swelling
Earlobes	Pinch marks
Upper lip and frenulum	Forced feeding, forced oral sex
Neck	Choke marks

Biting is considered to be a primitive type of assault and results when teeth are employed as a weapon in an act of dominance or desperation. Bite marks are usually associated with sex crimes, violent fights, and child abuse.[8]

Bite marks can be either by human or animals and its evidence is as important as finger prints, hair, blood, and semen samples. Differentiating factors between human and animal bite marks include the size, shape, contour, location, and color of the bite mark. A considerable knowledge on various animal dental patterns is required to differentiate between the two.

In the presence of clinical signs, such as ecchymosis, lacerations, and abrasions, on the victims in elliptical or ovoid pattern, a suspicion of bite marks should be considered. Bite marks may have a central area of ecchymoses (contusions) caused by 2 possible phenomena: (1) positive pressure from the closing of the teeth with disruption of small vessels or (2) negative pressure caused by suction and tongue thrusting. Bites produced by dogs and other carnivorous animals tend to tear flesh, whereas human bites compress flesh and can cause abrasions, contusions, and lacerations but rarely avulsion of tissue.[4]

## Reporting and documentation

Dentists who suspect or recognize some form of child abuse, have a responsibility of reporting it to the concerned authorities. Dental professionals are mandated by law to report suspicions of child abuse and neglect, but surveys show that dentists do not fulfil their obligation to report.[9] Courts have declared “any physician who fails to identify and report a child with historical, physical, and radiological findings that indicate abuse is guilty of professional negligence.”[10]

Whenever suspicions of abuse arise, a routine protocol should be followed, which includes questions about patient history and how the accident occurred, and all relevant information should be documented with radiographs, photographs, and impressions when necessary.[11] While many dentists report, the following are documented before reporting:

- The size, shape, location, color, degree of healing of the injury
- Detailed notes of behavioral indicators
- Pictures drawn of the injured area and labeled accordingly and radiographs of affected teeth
- Photographs of suspicious pathology
- Record of the date of consultation with other professionals

## Conclusion

When injuries are seen in cases with no proper history with severe signs and symptoms, the likelihood of abuse should be strongly suspected. Obtaining and maintaining a satisfactory history is important during initial examination procedure. At subsequent visits, the dentist must consider the changes in general health history as well as answers to specific questions. Dentists must become more aware of their moral, legal, and ethical responsibilities in recognizing and reporting child abuse and neglect.[12] Services and protection cannot be offered to the victim unless reported so it is our duty to recognize and report abuse. Some hurts cannot be fixed with band aid, so stop and fight abuse.
